# Characterisation of the clinical phenotype in Phelan-McDermid syndrome

**DOI:** 10.1186/s11689-021-09370-5

**Published:** 2021-07-10

**Authors:** Mónica Burdeus-Olavarrieta, Antonia San José-Cáceres, Alicia García-Alcón, Javier González-Peñas, Patricia Hernández-Jusdado, Mara Parellada-Redondo

**Affiliations:** 1 Department of Child and Adolescent Psychiatry, Institute of Psychiatry and Mental Health, Hospital General Universitario Gregorio Marañón, Calle Ibiza 43, 28009 Madrid, Spain; 2 IiSGM, Instituto de Investigación Sanitaria Gregorio Marañón, Madrid, Spain; 3 School of Psychology, Universidad Autónoma, Madrid, Spain; 4 School of Medicine, Universidad Complutense, Madrid, Spain; 5 CIBERSAM, Centro de Investigación Biomédica en Red Salud Mental, Madrid, Spain

**Keywords:** Phelan-McDermid syndrome, 22q13 deletion syndrome, SHANK3, Intellectual disability, Autism

## Abstract

**Background:**

Phelan-McDermid syndrome (PMS) is a rare genetic disorder compromising the 22q13 terminal region and affecting *SHANK3*, a gene crucial to the neurobehavioural phenotype and strongly linked to autism (ASD) and intellectual disability (ID). The condition is characterised by global developmental delay, ID, speech impairments, hypotonia and autistic behaviours, although its presentation and symptom severity vary widely. In this study, we provide a thorough description of the behavioural profile in PMS and explore differences related to deletion size and language ability.

**Methods:**

We used standard clinical assessment instruments to measure altered behaviour, adaptive skills and autistic symptomatology in sixty participants with PMS (30 females, median age 8.5 years, SD=7.1). We recorded background information and other clinical manifestations and explored associations with deletion size. We performed descriptive and inferential analyses for group comparison.

**Results:**

We found delayed gross and fine motor development, delayed and impaired language (~70% of participants non or minimally verbal), ID of different degrees and adaptive functioning ranging from severe to borderline impairment. Approximately 40% of participants experienced developmental regression, and half of those regained skills. Autistic symptoms were frequent and variable in severity, with a median ADOS-2 CSS score of 6 for every domain. Sensory processing anomalies, hyperactivity, attentional problems and medical comorbidities were commonplace. The degree of language and motor development appeared to be associated with deletion size.

**Conclusions:**

This study adds to previous research on the clinical descriptions of PMS and supports results suggesting wide variability of symptom severity and its association with deletion size. It makes the case for suitable psychotherapeutic and pharmacological approaches, for longitudinal studies to strengthen our understanding of possible clinical courses and for more precise genomic analysis.

**Supplementary Information:**

The online version contains supplementary material available at 10.1186/s11689-021-09370-5.

## Background

Phelan-McDermid syndrome (22q13 deletion syndrome or PMS), exhaustively explored by Phelan and McDermid [1], is a rare genetic disorder characterised by global developmental delay, hypotonia, intellectual disability (ID), severe speech impairments and autism spectrum disorder (ASD) to a variable degree [2]. Other manifestations include minor dysmorphic features and somatic conditions, especially gastrointestinal reflux and renal problems [3, 4]. Sometimes additional neurological problems occur, including seizures, reduced pain and temperature sensitivity or structural and functional brain abnormalities [3, 5]. Researchers also report a high prevalence of psychiatric comorbidities and developmental regression [6, 7]. The exact prevalence of the syndrome remains unknown, and the condition is likely underdiagnosed, due to its unspecific clinical phenotype and the need for molecular genetic testing to establish a diagnosis [8]. Recent descriptions of this syndrome reveal several clinical features, such as ASD and ID, attention deficit, hyperactivity and impulsivity, aggressive behaviour, language impairments and medical comorbidities [4, 9].

The association between the *SHANK* gene family and neurodevelopmental disorders is well established [10–12]. Some researchers consider the loss of a functional copy of the *SHANK3* gene, which is responsible for the codification of a scaffolding protein in the postsynaptic density, the cause of the main clinical features of PMS [3, 13]. Recent studies suggest that deletions or mutations compromising *SHANK3* can cause a monogenic form of both ASD and ID, accounting for around 1% of the overall ASD cases [8, 14] and 0.3–1% of the ID [15, 16]. Researchers have explored genotype-phenotype associations, showing that deletion size and locus are often correlated with speech abilities [3, 17], hypotonia and dysmorphic features [13, 18] and adaptive skills [9, 19]. Findings concerning other traits, such as autistic or aggressive behaviour, are inconsistent [14, 17, 20]. In this study, we aim to conduct a deep phenotypic characterisation of the clinical and neurobehavioural traits of a large national sample of participants with Phelan-McDermid syndrome, using standardised, robust instruments to assess repetitive or otherwise altered behaviours, hyperactivity, attentional problems, sensory processing difficulties, global adaptive functioning and autistic features. Specifically, we want to explore the similarity of the behavioural profiles between verbal and non-verbal individuals. Therefore, our research questions read: *#1 Are the characteristics of our PMS sample like other cohorts described? #2 How is language ability related to other phenotypic characteristics? Do the clinical profiles of verbal and non-verbal individuals differ?*

We expect our findings to be in accordance with those previously reported in the aforementioned clinical descriptions, including developmental and language delay, presence of ASD traits and challenging behaviours (e.g. aggression, hyperactivity), as well as certain correlations between these characteristics and the size of the deletion.

## Methods

### Participants

All individuals from the Spanish Phelan-McDermid Association (89 families) and also patients with an identified *SHANK3* alteration (1 family) assisted in the regional AMITEA programme (Comprehensive Medical Care-Autism Spectrum Disorders) [21] of the Hospital General Universitario Gregorio Marañón in Madrid through the end of 2018 were invited to participate. Sixty children and adults (50% female), between 11 months and 41 years of age (M=10.6 years, median=8.5, SD=7.1), participated (response rate=66.7%). Sample characteristics are in Table 1.

**Table 1:** Main sociodemographic and clinical characteristics of the sample

Sample characteristics	N	%
Sex		
Male | Female	30 | 30	50 | 50%
Age		
0–5 years	14	23.3%
6–11 years	25	41.7%
12–17 years	12	20.0%
≥18 years	9	15.0%
Socioeconomic status		
Low | M-low | Middle	1 | 14 | 9	1.6% | 23.3% | 15%
M-upper | Upper	17 | 19	28.3% | 31.6%
Gestational age		
Pre-term (<37 weeks)	12	20.0%
Full-term (38–42 weeks)	48	80.0%
Language (≥3 yo)*	**54**	
No words	28	51.9%
Single words	7	13.0%
Simple sentences	11	20.4%
Fluid speech	8	14.8%
Developmental regression/Loss of skills (parent reported)		
Yes | No	25 | 35	41.7 | 58.3%
Recovered skills		
Yes | No	12 | 13	48 | 52%
**Autistic and other clinically relevant behaviours**	**N**	**%**
ADOS-2*	48	-
Total-CSS 1–3	12	25.0%
Total-CSS 4–5	8	16.7%
Total-CSS 6–10	28	58.3%
CBCL* (1.5–5 years | 6–18 years)	10 | 28	
Emotionally reactive	4 | n/a	40% | n/a
Anxious/depressed	0 | 0	0% | 0%
Somatic complaints	0 | 6	0% | 21%
Sleep problems	1 | n/a	10% | n/a
Attention problems	6 | 20	60% | 71%
Withdrawn	7 | 7	70% | 25%
Aggressive	3 | 6	30% | 21%
Rule-breaking	n/a | 3	n/a | 11%
Social problems	n/a | 18	n/a | 64%
Thought problems	n/a | 15	n/a | 54%
SDQ*	33	
Emotional symptoms	1	3.0%
Conduct problems	2	6.1%
Hyperactivity	21	63.6%
Peer relations difficulties	22	66.7%
Difficulties with prosocial behaviour	28	84.8%
Total	17	51.5%

*Language: We used 3 years as the developmental threshold for language acquisition, and only considered participants over this age for descriptive purposes to better distinguish delay in acquisition from absence of speech. The remaining 6, however, were minimally verbal. *ADOS-2 (Autism Diagnostic Observation Scale-2): Calibrated severity scores indicate severity of autistic symptoms. *CBCL (Child Behavior Checklist): One version for children aged 1.5 to 5 years and another for children aged 6 to 18 with five shared subscales; values indicate individuals who scored over the cutoff for borderline clinical relevance. *SDQ (Strengths and Difficulties Questionnaire): Values indicate individuals who scored over the cutoff for borderline clinical relevance

The only inclusion requirement was having a diagnosis of Phelan-McDermid syndrome. Trained psychologists and psychiatrists conducted the assessments at Gregorio Marañón hospital. Caregivers gave informed consent. The hospital ethics committee approved the study.

### Materials and assessment

- Autistic symptomatology was quantified with the following instruments: *Autism Diagnostic Observation Schedule–2* (ADOS-2 [22]), *Autism Diagnostic Interview–Revised* (ADI-R [23]), *Repetitive Behaviours Scale–Revised* (RBS-R [24];), *Social Responsiveness Scale* (SRS [25];) and *Sensory Profile-2* (SP-2 [26];)*.* ADOS-2 is a semi-structured, standardised assessment of communication, social interaction, play and restricted and repetitive behaviours; we calculated ADOS-2 calibrated severity scores (CSS) [27] to offer a comparable measure of ASD severity across ages and modules. ADI-R is an extensive interview conducted with caregivers of individuals suspected of having ASD, which provides a thorough assessment of early development in three domains: reciprocal social interactions (A); language/communication (B); and restricted, repetitive and stereotyped behaviours and interests (C). Both ADOS-2 and ADI-R are gold-standard instruments for the diagnosis of ASD and in combination have proven to offer a reliable assessment of autistic symptomatology; however, their validity in samples with severe to profound ID is decreased [28]. In this work, ADOS-2 and ADI-R scores are used to quantify autistic symptoms, and no clinical diagnosis of autism is intended. ADI-R items 11–28, which define regression as a loss of consistently acquired skills during at least 3 months, provided information about regression. Parents completed the parent-rated RBS-R, which assesses repetitive behaviours and the parent-rated SRS, which measures the severity of autistic symptoms in natural settings for verbal individuals, along with the SP-2 to assess children’s sensory processing systems and patterns.
- Adaptive behaviour was evaluated by means of the *Vineland Adaptive Behavior Scales-3* (VABS-3) in its comprehensive parent/caregiver form [29]. This form was used and not the interview due to time constraints. These scales have been a leading instrument for supporting the diagnosis of intellectual disabilities for the last decades, providing scores for communication, daily living skills, socialization and motor skills domains, as well as an overall adaptive behaviour composite. Motor domain only considers children younger than 10 years. The VABS-3 has a mean standard score of 100 and SD of 15 for domains and a mean v-score of 15 and SD of 3 for subdomains.
- Other behavioural difficulties were assessed through the following: (i) *Aberrant Behavior Checklist—Community* (ABC-C [30]), a checklist for assessing problem behaviours of children and adults with developmental disabilities; (ii) *Child Behavior Checklist* (CBCL [31]), a report for identifying problem behaviours in children aged 1.5–5 and 6–18 years; and (iii) *The Strengths and Difficulties Questionnaire* (SDQ [32]), a brief screening questionnaire for children aged 4–17. Both the CBCL and SDQ offer cutoff scores for clinically relevant and borderline altered behaviours. To account for all altered behaviour, we considered scores above the borderline cutoff.
- Background information regarding early development, medical comorbidities, previous neurophysiological, imaging procedures, etc. was also gathered using a self-devised interview with parents. We used the *Cumulative Illness Rating Scale* (CIRS) [33] in a retrospective manner to obtain information on medical events over participants’ lifetimes.
- Genetic information was provided by parents and caregivers through existing clinical genetic reports. We only used deletion size. Genetic tests leading to diagnosis included different assessments such as Karyotype, FISH, MLPA and array-CGH.

### Data analysis and cluster analysis

Study data were stored and managed using REDCap (Research Electronic Data Capture) electronic data capture tools hosted at Instituto de Investigación Sanitaria Gregorio Marañón. REDCap is a secure, web-based application designed to support data capture for research studies [34].

We performed descriptive and inferential analyses, including unpaired T-tests and one-way ANOVA for group comparisons of continuous variables, Chi-square tests to compare proportions and Pearson’s correlation coefficients to explore associations between behavioural variables and deletion size. For those variables with non-normal distributions, we performed non-parametric statistical tests, including Mann-Whitney U tests for group comparisons of continuous variables, Wilcoxon signed-rank test for paired comparisons and Spearman rank correlation coefficients. We computed 95% CI with bootstrapping (BCa 95% CI) for the parameter estimates and reported the 95% CI of mean differences for parametric tests. We used PASW Statistics 18 [35] for statistical analysis and R-3.6.3 [36] to generate graphs.

Ward’s hierarchical cluster analysis was performed on 42 individuals using the following variables: deletion size, sex, developmental regression, language status and ADOS-2 total CSS score to explore grouping of clinical variability.

## Results

### Sample description

Participants (n=60) were predominantly younger than 18 years (age ranging from 0.92 to 23 years, one individual aged 41 years; median=8.54, SD=7.1), and sex distribution was even. Sample characteristics are summarised in Table 1.

Forty-eight participants had ADOS-2 scores: 46 administered during this study, 1 administered 2 years earlier by a research-trained professional in AMITEA (same module) and 1 from a private report from the previous year. The unassessed cases (n=12) either showed important mobility limitations and severe ID (estimated MA<15mo) or lived too far away to visit. Module 1 was used for 35 participants, module 2 for 7, module 3 for 5 and module T for 1.

### Clinical findings

#### Early development

Average age at independent walking was 22 months (SD=8.2, range=[11-42]); 4 participants older than 42 months (7.7%) could not walk at the time of the assessment. Thirty-two participants showed delayed walking, defined as walking unaided after 18 months of age, and at least 21 had not crawled. Overall, 54 (90%) had delayed fine and/or gross motor development.

Considering only participants aged 3 and up, 28 (51.9%) were non-verbal, 7 (13.0%) used single words, 11 (20.4%) used simple sentences and 8 (14.8%) used complex sentences or fluid speech. Age at acquisition of language ranged from 8 months to 6.5 years for words and from 2.5 to 14 years for sentences. On average, participants produced their first words at ~32 months and first sentences at ~6 years. For descriptive purposes, we divided the sample in two groups, “Non-verbal or minimally verbal”, used interchangeably for subjects producing none to single words and “Verbal” for those who use sentences.

#### Developmental regression

Parents reported a history of developmental regression in 24 cases and an additional case of “loss of skills”. Of these, only 6 met the strict ADI-R criteria for language regression. However, in 17 cases, parents reported a noticeable loss of skills in speech production (i.e. abruptly stopped babbling or using acquired words), regardless of initial language level, and 21 reported regression or loss of skills in other domains: social engagement and reciprocity (21), motor skills (6), self-help skills (6) and play (3). In at least 4 cases, regression was possibly related to medical causes (mononucleosis, severe bronchitis, severe bronchospasms, metabolic acidosis).

Fourteen participants experienced regression before or at 30 months (mean=48.7, range=[11–168] months). Notably, 4 cases occurred at age 10 and older: One involved regression in speech production with regained abilities; another was related to language articulation; the remaining 2 experienced a generalised regression with emotional dysregulation at ages 13 and 14.

Of the 25 cases, ten individuals regained a similar level of functioning after times ranging from 5 months to 9 years and 2 regained some of the abilities (6 out of 7 verbal participants and 6 out of 18 non-verbal participants). Thirteen participants (22%) experienced a persistent loss of skills. We explored possible associations between regression and sex (*X*^*2*^=.069, *p*=.793, V=.034), language level (*X*^*2*^=.396, *p*=.529, V=.087, age≥3years old) and deletion size (*t*(51)=1.324, *p*=.191, Hedge’s *g=0.369*).

### Autism profile

For the whole sample, median ADOS-2 CSS was 6 for SA, RRB and total score. Table 2 shows ASD symptomatology separated by language. ADOS-2-CSS and ADI-R A and C mean scores were similar or slightly higher for minimally verbal individuals, although with considerable CI overlap.

**Table 2:** ASD symptomatology by language level

		Verbal		Minimally verbal (≥3yo)		
		***n***	Mean (SD) [range]	***n***	Mean (SD) [range]	Comparison
ADOS-2	SA-CSS	18	5.50 (*2.33*) [1-10] BCa 95% CI [4.44, 6.61]	27	5.56 (*2.78*) [2-10] BCa 95% CI [4.32, 6.71]	*U=*229.500, *Z=-*.316, *p=*.752, η^2^*=*0.002
	RRB-CSS		5.11 (*2.59*) [1-10] BCa 95% CI [4.00, 6.22]		6.22 (*1.48*) [1-9] BCa 95% CI [5.58, 6.80]	*U=*174.000, *Z=*−1.656, *p=.*098, η^2^*=*0.062
	Total-CSS		5.00 (*2.17*) [1-9] BCa 95% CI [3.89, 6.09]		5.22 (*2.31*) [2-10] BCa 95% CI [4.32, 6.15]	*U=*230.500, *Z=*−.297, *p=*.766, η^2^*=*0.002
ADI-R	A	18	16.33 (*6.71*) [6-28] BCa 95% CI [13.54, 19.24]	32	20.94 (*5.84*) [6-30] BCa 95% CI [18.91, 22.91]	*U=*173.500, *Z=-*2.320, *p=*.020, η^2^*=*0.108
	B		13.44 (*5.68*) [2-23] BCa 95% CI [10.85, 16.06]		12.25 (*3.06*) [0–14] BCa 95% CI [11.01, 13.19]	n/a
	C		4.44 (*2.64*) [0–9] BCa 95% CI [3.33, 5.45]		4.62 (*2.34*) [0–8] BCa 95% CI [3.84, 5.49]	*t*(48)=.250, 95% CI [−1.27, 1.63], *p*=.803
SRS	Total	11	87.27 (*35.00*) [26–141] BCa 95% CI [66.76, 106.88]	n/a		
	Social Awareness		10.82 (*4.26*) [4-19] BCa 95% CI [8.73, 13.00]			
	Social Cognition		18.36 (*6.07*) [9-26] BCa 95% CI [14.79, 21.82]			
	Social Communication		28.00 (*13.02*) [4-46] BCa 95% CI [19.69, 34.95]			
	Social Motivation		13.91 (*7.11*) [4-28] BCa 95% CI [10.18, 17.62]			
	Autistic Mannerisms		15.27 (*7.59*) [4-25] BCa 95% CI [10.73, 19.73]			
RBS-R	Stereotyped	15	1.67 (*2.47*) [0–8] BCa 95% CI [.60, 2.80]	28	3.32 (*3.35*) [0–12] BCa 95% CI [2.19, 4.67]	*U*=141.500, Z=−1.790, *p=*.073, η^2^*=*0.075
	Self-injurious		1.60 (*1.59*) [0–4] BCa 95% CI [.87, 2.27]		1.89 (*2.20*) [0–8] BCa 95% CI [1.21, 2.69]	*U*=208.000, Z=−.053, *p=*.958, η^2^*<*0.001
	Compulsive		4.33 (*6.62*) [0–20] BCa 95% CI [1.69, 7.27]		2.14 (*2.92*) [0–10] BCa 95% CI [1.17, 3.40]	*U*=180.000, Z=−.792, *p=*.429, η^2^*=*0.015
	Ritualistic		4.33 (*4.34*) [0–14] BCa 95% CI [2.41, 6.45]		1.54 (*2.22*) [0–7] BCa 95% CI [.77, 2.48]	*U*=111.500, Z=−2.614, *p=*.009, η^2^*=*0.159
	Sameness		6.53 (*7.61*) [0–27] BCa 95% CI [3.45, 10.22]		3.46 (*4.46*) [0–13] BCa 95% CI [1.72, 5.39]	*U*=155.000, Z=−1.435, *p*=.151, η^2^*=*0.048
	Restricted		1.73 (*3.33*) [0–12] BCa 95% CI [.47, 3.27]		1.68 (*2.31*) [0–8] BCa 95% CI [.96, 2.55]	*U*=187.000, Z=−.632, *p=*.527, η^2^*=*0.009
SP-2	Auditory	10	18.90 (*5.63*) [13-31] BCa 95% CI [15.90, 22.56]	22	19.45 (*5.57*) [9-30] BCa 95% CI [17.23, 21.73]	*t*(30)=.260, 95% CI [−3.80, 4.91], *p*=.796
	Visual		11.80 (2.57) [7-15] BCa 95% CI [10.30, 13.30]		11.50 (4.70) [4-22] BCa 95% CI [9.59, 13.42]	*U*=89.500, Z=−.838, *p=*.411, η^2^=0.022
	Touch		25.40 (5.76) [16-36] BCa 95% CI [22.10, 28.60]		24.82 (7.80) [14-42] BCa 95% CI [21.75, 28.18]	*t*(30)= −.210, 95% CI [−6.23, 5.06], *p*=.835
	Movement		19.40 (5.04) [11-29] BCa 95% CI [16.60, 22.11]		21.45 (7.13) [5-32] BCa 95% CI [18.14, 24.37]	*t*(30)=.820, 95% CI [−3.06, 7.17], *p*=.419
	Body position		18.10 (5.82) [8-27] BCa 95% CI [14.80, 21.60]		18.86 (5.85) [9-29] BCa 95% CI [16.27, 21.55]	*t*(30)=.343, 95% CI [−3.79, 5.31], *p*=.734
	Oral		15.40 (3.13) [13-21] BCa 95% CI [13.80, 17.20]		16.95 (4.51) [12-27] BCa 95% CI [15.31, 18.78]	*U*=89.000, Z=−.860, *p=*.411, η^2^=0.023
	Seeking		44.00 (8.62) [28–55] BCa 95% CI [39.00, 49.07]		45.18 (12.51) [22–72] BCa 95% CI [40.08, 50.58]	*t*(30)=.270, 95% CI [−7.76, 10.13), *p*=.789
	Avoiding		47.50 (13.79) [23–71] BCa 95% CI [39.30, 55.70]		37.59 (8.06) [19–51] BCa 95% CI [33.95, 41.11]	*t*(30)= −2.566, 95% CI [−17.80, −2.02], *p*=.016
	Sensitivity		43.30 (6.73) [32–54] BCa 95% CI [39.50, 47.20]		41.36 (9.67) [26–65] BCa 95% CI [37.64, 45.71]	*t*(30)= −.571, 95% CI [−8.86, 4.99], *p*=.572
	Registration		54.40 (13.01) [35–76] BCa 95% CI [46.90, 61.93]		53.95 (14.32) [33–78] BCa 95% CI [47.72, 60.51]	*t*(30)= −.084, 95% CI [−11.30, 10.41], *p*=.934
	Conduct		21.70 (4.60) [15-31] BCa 95% CI [19.30, 24.20]		19.18 (7.06) [9-30] BCa 95% CI [16.29, 22.35]	*t*(30)= −1.029, 95% CI [−7.52, 2.48], *p*=.312
	Social-emotional		36.50 (8.70) [22–50] BCa 95% CI [31.50, 41.43]		27.82 (7.23) [15-42] BCa 95% CI [24.80, 30.68]	*t*(30)= −2.957, 95% CI [−14.68, −2.69], *p*=.006
	Attentional		28.60 (7.43) [15-38] BCa 95% CI [24.20, 32.70]		26.86 (7.45) [15-42] BCa 95% CI [24.11, 30.00]	*t*(30)= −.612, 95% CI [−7.53, 4.06], *p*=.545

*No correction for multiple comparisons. Only participants aged ≥3 years old.* ADOS-2: social affect (SA), repetitive and restrictive behaviours (RRB), calibrated severity score (CSS). CSS range 1–10 indicating increasing severity. ADI-R: Only participants aged 4 and up. Qualitative alterations of reciprocal social interactions (A), range 0–30; qualitative alterations of language/communication (B), non-verbal range 0–14, verbal range 0–26; restricted, repetitive and stereotyped behaviours and interests (C), range 0–12. SRS (Social Responsiveness Scale), only verbal participants: total range 0–195, social awareness range 0–24; social cognition range 0–36, social communication range 0–66, social motivation range 0–33, autistic mannerisms range 0–36. RBS-R (Repetitive Behavior Scale-Revised): stereotyped range 0–18, self-injurious range 0–24, compulsive range 0–24, ritualistic range 0–18, insistence in sameness range 0–33, restricted range 0–12. SP-2 (Sensory Profile-2): sensory sections: auditory range 0–40, visual range 0–30, touch range 0–55, movement range 0–40, body position range 0–40 and oral range 0–50; sensory quadrants: seeking range 0–95, avoiding range 0–100, sensitivity range 0–95 and registration range 0–110; behavioural section: conduct range 0–45, social-emotional range 0–70 and attentional range 0–50. Confidence interval for the group means calculated with bootstrapping (95% BCa CI). 95% CI for the mean difference calculated only for normally distributed variables

#### Social affect and social interaction

Social affect scores as measured by the ADOS-2 SA-CSS were similar for verbal and non-verbal participants but seemed to be higher (although with CI overlap) for non-verbal individuals in the ADI-R domain A: reciprocal social interaction (see Table 2).

#### Repetitive and restrictive behaviours

Scores in the ADOS-2 RRB-CSS and the ADI-R domain C were similar for verbal and non-verbal participants (see Table 2). Within the RBS-R questionnaire, verbal individuals appeared to score higher than non-verbal in the ritualistic subscale (*U*=111.500, Z=−2.614, *p*=.009, η^2^=0.159).

#### Sensory processing difficulties

Scores from the SP-2 separated by language level can be found in Table 2. Considering all participants, at least 60% scored over percentile 80 in the sensory systems “Touch”, “Movement” and “Body position”, accounting for sensory processing difficulties such as increased reactivity to tactile stimuli, search of abrupt vestibular input, low muscle tone and increased pain/temperature tolerance—45% reported a high pain threshold. Other areas, such as oral or visual processing, were not particularly affected. With regards to sensory patterns, over 80% showed impairments in registration (i.e. ignoring or failing to perceive certain stimuli, e.g. seeming disconnected from the environment, having a high pain threshold or losing eye contact in sustained interaction), and over 60% reported attentional problems, such as poor attention span and being easily distracted. Detailed information is presented in Supplementary table 1.

### Adaptive functioning

**Fig. 1:**
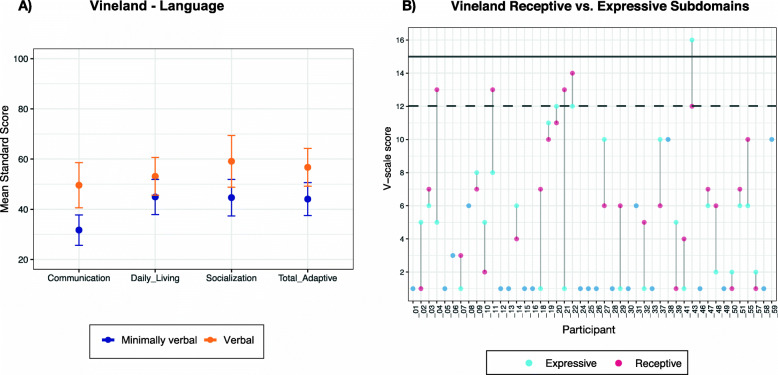
VABS-3 standard scores by language level and receptive and expressive scores for every participant. **A** Differences based on language (only participants over age 3 considered): communication (*U=84.500, Z= −3.146, p=.002*), daily living (*t(40)= −1.561, 95% CI [−18.84, 2.42], p=.126*), socialization (*t(40)=-2.432, 95% CI [−26.49, −2.44], p=.020*), total adaptive (*t(40)= −2.510, 95% CI [−22.85, −2.47], p=.016*). “Verbal” n=15; “Minimally verbal” n=27. Error bars represent confidence interval of the mean. **B** Scores in receptive and expressive subdomains for each participant. Solid and dashed lines represent population mean and SD, respectively

We obtained results for 46 participants (all ages considered; characteristics of participants missing VABS were similar to completers). Mean (*SD*) scores from the VABS-3 domains were communication 39.28 (*18.62*), daily living skills 49.15 (*17.90*), socialization 51.37 (*19.72*) and total adaptive composite 49.87 (*16.94*). V-scale scores and age equivalents (AE) were largely below normative values (medians reported): receptive 4 (AE 16.50mo), expressive 2 (AE 9.50mo), written 1 (n/a), personal 4.5 (AE 23mo), domestic 7 (n/a), community 3.5 (n/a), interpersonal 6 (AE 15.50mo), play 6 (AE 15.50), coping 6 (n/a), gross motor 8 (AE 20mo) and fine motor 5 (AE 17mo). Regarding language, V-scale scores for receptive and expressive subdomains fell within the normative ranges for 5 and 3 participants, respectively. To further look at these communicative skills, we explored possible intra-subject differences between scores in receptive and expressive subdomains (*Z=*−1.100, *p=*.271, Wilcoxon’s test). Results showing receptive and expressive V-scale scores and domain standard scores separating individuals by their language level are in Fig. 1.

In line with other findings [9], age seems to play a role in adaptive behaviour. Younger individuals showed higher standard scores, and age was inversely correlated with every VABS-3 domain (communication: *r*_*s*_(44)= −.296, *p=*.046; daily living: *r*(44) =−.512, *p<*.001; socialization: *r*(44)= −.421, *p=*.004; total adaptive: *r*(44)= −.726, *p=*.001), a result compatible with the concept of “growing into deficit”, i.e. increasing discrepancy with age-expected performance.

### Associated symptoms

#### Behavioural alterations

**Fig. 2:**
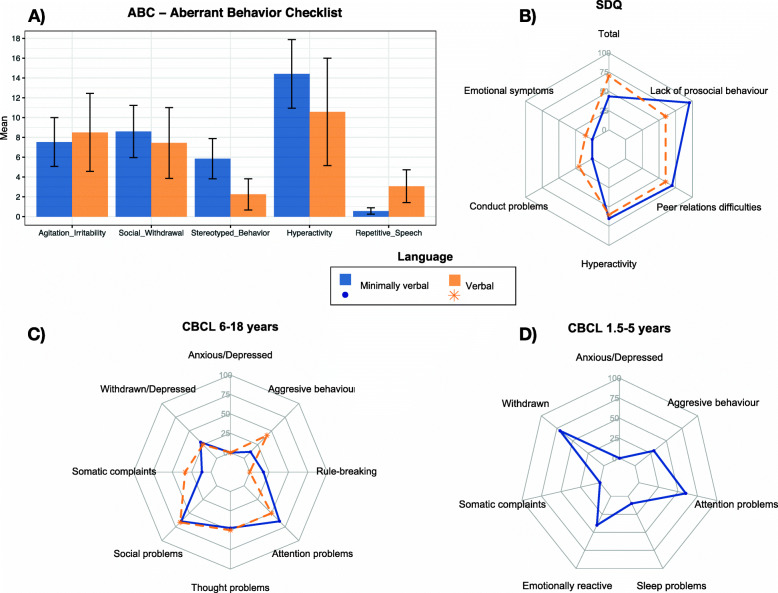
Behavioural alterations reported in ABC, SDQ and CBCL. **A** ABC (Aberrant Behavior Checklist), “Verbal” n=16, “Minimally verbal” n=28. Error bars represent confidence interval of the mean. **B** SDQ (Strengths and difficulties Questionnaire), “Verbal” n=10, “Minimally verbal” n=23. Percentage of participants over the borderline cutoff for each subscale. **C** CBCL for ages 6–18 years, “Verbal” n=12, “Minimally verbal” n=17. Percentage of participants over the borderline cutoff for each subscale. **D** CBCL (Child Behavior Checklist) for ages 1.5 to 5 years, n=10, all minimally verbal. Percentage of participants over the borderline cutoff for each subscale

Aggressive behaviours were present in 23.7% of the sample as measured by the CBCL. The ADI-R also recorded some degree of aggressive behaviour in 35.8% of individuals across lifetime, while 52.8% had self-injurious behaviours. The CBCL and SDQ captured clinically relevant scores for hyperactivity, attentional problems and difficulties relating to peers and establishing social contact and appear in Fig. 2 as percentage of individuals complying with borderline/clinical scores. In SDQ, verbal participants showed more prosocial behaviour than non-verbal individuals (5.40 vs. 2.87; *U=*53.500, *Z=−2.443*, *p=.015*, Hedges’ *g=*0.984), whereas there were no apparent differences in respect to language in the CBCL. Within the ABC-C, the largest differences between verbal and >3 years old, non-verbal participants were found for stereotyped behaviour and repetitive speech. Scores can be found in Supplementary table 2.

#### Medical comorbidities

The most widespread conditions, reported in 53 cases, were musculoskeletal, especially hypotonia and bone anomalies such as claw feet and kyphosis. Recurrent respiratory tract, ear or urinary tract infections were reported in 45 cases, and usually struck hardest during the first 4 years of life, although they persisted in some cases. Two individuals suffered meningitis within 4 months postnatally. Thirty-five participants suffered from lower gastrointestinal disturbances (mainly constipation/diarrhoea) and 30 from gastroesophageal reflux. Thirty-three had neurologic findings of varying relevance, of which 7 had epilepsy and 6 a history of febrile seizures or epileptic-like episodes. Respiratory problems were common among 30 participants. Twenty-four individuals showed food intolerances, especially to lactose and legumes, and 5 had food allergies. Important sleep difficulties appeared in 19 individuals, including difficulty falling asleep, recurrent waking up or short sleeping times. Eighteen patients had various renal anomalies, including asymmetric or duplex kidneys, and 11 had genitourinary tract complaints, such as recurrent infections, urethral reflux or hypospadias. Sixteen participants had skin complaints, the most common being atopic dermatitis. Fifteen participants reported low iron levels. Fourteen had cardiac anomalies. Ten had endocrine-metabolic problems. Supplementary tables 3 and 4 summarise the reported medical comorbidities using CIRS items of any severity and further medical history. Results are retrospective and encompass events occurring at any point during the participants’ lifetime.

#### Genetic information and cluster analysis

Genetic information including deletion size was available for 53 participants (52 aCGH and 1 MLPA with precise estimated loss); another exome sequencing test reported a point mutation affecting *SHANK3*. The remaining 6 lacked deletion size information due to the type of test (1 karyotype, 2 FISH, 3 MLPA). The reported information also identified 4 ring chromosomes, 6 mosaics ranging from 35 to 70% and 15 individuals with additional CNVs.

**Fig. 3:**
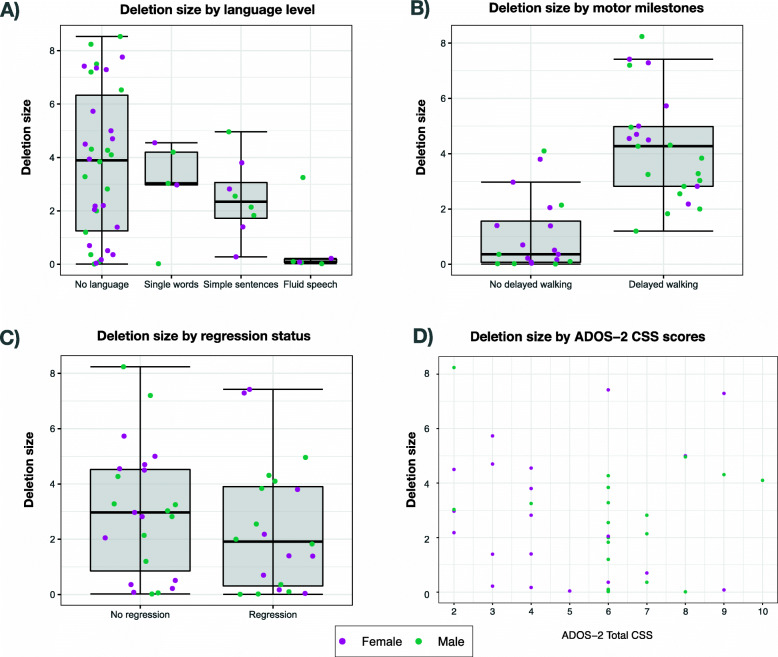
Differences in sample characteristics and 22q13 deletion sizes. **A** Deletion size and language level. “No language” n=34; “Single words” n=5, outlier experienced a regression during puberty; “Simple sentences” n=8; “Fluid speech” n=6, outlier is a mosaic. **B** Deletion size and motor milestones. “No delayed walking” n=23; “Delayed walking” n=28. **C** Deletion size and regression status. “No regression” n=31; “Regression” n=21. **D** Deletion size and ADOS-2 Total CSS scores

We tested the association of deletion size with language, motor milestones, regression and ADOS-2 CSS score (Fig. 3). Participants with delayed motor milestone acquisition (i.e. delayed walking) apparently had larger deletion sizes than those who walked at a typical age (*t*(49)=−6.147, 95% CI [−4.25, −2.16], *p*<.001), and so did participants with absence of language compared to those with fluid speech (*F*(3,49)=3.012, *p=*.039). Detailed information on deletion size and its association with phenotypic variables are presented in Supplementary table 5.

To explore similarities within the phenotype, we conducted an exploratory hierarchical cluster analysis on 42 participants using clinical and demographic data. This resulted in four groups for which the clearest separation was the deletion size together with ADOS-2 CSS scores. Cluster 1 was composed of 11 participants with deletions of medium-to-large size (M=4.16Mb(*1.70*)) and low CSS scores (median=3, range=[2-4] ). Cluster 2 had 6 participants with relatively large deletions (M=5.51Mb(*1.47*)) and high CSS scores (median=8.5, range=[6-10] ). Cluster 3 was formed by 10 individuals with mean deletion size of 2.60Mb(*.96*) and accompanying autistic symptoms (median=6, range =[6-7]) and cluster 4 gathered subjects with the smaller deletions (M=.35Mb(.*46*)), varying autistic symptom severity (median=6, range=[3-9]), and the lowest proportion of participants with delayed motor milestones. Distribution of clinical variables and sex within clusters is shown in Supplementary Figure 1 and Supplementary table 6.

## Discussion

This is the first general phenotypic description of a representative cohort of Spanish PMS patients. We evaluated sixty patients (85% of them children and adolescents) with an even sex distribution. Participants’ independent walking and language acquisition were delayed. Forty-two percent of participants experienced development regression or loss of skills, with half of them recovering skills over varying periods. Adaptive functioning ranged from very to moderately low. Autistic symptomatology was variable in type and severity, with a median ADOS-2 CSS of 6 for every domain. Caregivers often reported that participants experienced sensory problems and attention and hyperactivity difficulties, as well as medical comorbidities, such as gastrointestinal issues, respiratory infections, neurological anomalies and sleep difficulties.

### Developmental milestones

Our findings align with previous descriptions of patients with PMS. With regards to developmental milestones, our data suggest important motor delay in up to 90% of the sample and generalized language impairment, with 14.8% of participants using fluid speech, comparable with other studies [3, 4, 14, 37]. There were no apparent differences between receptive vs. expressive communicative skills, although results showed wide variability both within and between subjects.

### Adaptive skills, ASD and regression

Per the VABS-3, all participants functioned below their chronological age, as in previous studies [14, 24], with scores two or more SD below average, suggesting that individuals with PMS, regardless of autistic symptomatology, may be in the moderate to severe range of ID. Adaptive behaviour is necessary to determine the level of ID, and ID status can be granted on the basis of a delayed adaptive level in relation to chronological expectations. Low adaptive skills are therefore part of the mental health classification systems (i.e. DSM and CIE) and a good index to determine the presence of ID [29].

However, less affected individuals with PMS may go undetected because genetic testing is likelier for individuals with low IQ or dysmorphic features. Despite the generalised difficulties in the communication domain, our group results are mostly in line with the patterns observed in ID, with higher socialization scores. A more exhaustive profile of adaptive skills taking into account relevant variables, such as age, degree of ID or autistic symptoms, is warranted.

The presence of autistic symptoms in PMS is largely acknowledged; nevertheless, the estimated prevalence of ASD in PMS remains disputed, with the highest figures pointing towards more than 80% [14], but with a broad range, that likely depends on the diagnostic tools used. Some authors have suggested that PMS may have a different autistic-like profile than strictly described by DSM-IV [14, 38] or even posit that the characteristics of PMS may be related to communication impairments rather than an autistic manifestation [39]. Our results show a range of behaviours compatible with ASD, including sociocommunicative difficulties, resistance to change, unusual or restricted interests and stereotyped and ritualistic behaviour. Interestingly, verbal participants showed higher scores in subscales measuring ritualized behaviours, more connected to insistence in sameness (IS) than repetitive sensorimotor actions (RSM) within RRBs, and might be associated with less social impairment, while RSM appear more strongly associated with cognitive ability [40]. Sensory processing anomalies were also widespread, especially those related to tactile and proprioceptive stimuli. Nevertheless, these results must be interpreted with care, as even gold-standard instruments show suboptimal validity in populations with profound ID [41]. Categorical assessment in such populations is challenging, but the inclusion of clinical judgement would likely refine these estimates and more accurately inform about the prevalence of autism in a sample like this. While it was not the intention of this work to provide a clinical diagnosis of autism, we understand that this may be necessary for families in particular contexts to be granted services.

Researchers should integrate robust standardised tools, direct observation and clinical evaluation by experienced professionals to explore and describe more deeply the ASD profile in PMS. This would help account for potential differences with idiopathic autism or other forms of syndromic autism and discern whether these manifestations correspond to a distinct ASD profile.

Concerning developmental regression, we identified that half of the 42% of participants that had an apparent loss of abilities regained previous functioning. The percentage of participants with permanent deficits (22%) we found is in line with previous findings of 26–28% [7, 14]. The most affected areas for our sample were communication/language and social engagement, while other areas, such as self-help or motor skills, were not reported as often as in previous studies. Nevertheless, the assessment tools may not detect regression in children with very low abilities. Some patients experienced a very specific global regression during early puberty or adolescence, an interesting finding worth follow-up investigation.

### Clinical implications of behavioural problems and medical comorbidities

As in previous studies, hyperactivity and attention deficit were prevalent across all tools [3, 14, 42]. Although these symptoms are commonly observed in ID and may not be specific to PMS, they merit attention. Aggression and self-injurious behaviour were problematic for one third and half of the sample, respectively, so interventions aimed at improving not only communication, but also self-regulation strategies are desirable. Clinically relevant social withdrawal and internalising emotional symptoms were rare, as reported by parent questionnaires, which highlights the difficulty of evaluating internalising symptomatology in this population and a need for appropriate tools. However, mood and behavioural symptoms and psychiatric decompensation have been previously reported and systematically reviewed [43]. This exposes the importance of accurate and prompt assessment of changes in the clinical course, including mood lability and hypo- or hyperthymic profiles.

Similar to previous work [44], the most common medical comorbidities included gastrointestinal disturbances and gastroesophageal reflux, respiratory infections, food intolerances, sleep disturbances, skin complaints, genitourinary or renal anomalies, and epilepsy or febrile seizures, along with recurrent paediatric infections. It is of utmost importance to monitor the health of these patients, particularly in the context of their difficulties communicating pain or distress. Health status impacts behaviour, sleep and well-being in general, and therefore developmental and educational opportunities. Health care professionals need training in recognising and diagnosing medical comorbidities in patients with severe ID.

### Genotype-phenotype associations and hierarchical clustering

Regarding phenotype association with deletion size, we found mixed results across several traits. For example, motor development and language were less affected in individuals with smaller deletions, and we found no association with developmental regression or epilepsy, as previously reported [7, 20]. There was no clear association with ADOS or ADI-R or other behavioural manifestations, such as aggression or hyperactivity [4]. Nevertheless, our study included only reported information stemming from different genetic tests and resolution levels. A deeper analysis resulting from more precise genetic testing could help identify therapeutic targets and elucidate the relationship between these patients’ development and specific clinical outcomes and their genomes.

Our cluster analysis yielded four groups on the basis of deletion size and autistic symptom severity. While certain selected characteristics were apparently grouped in a congruent fashion with previous findings (e.g. motor delay), others were not (e.g. language status), which is surprising considering that deletion size was associated with language ability. More fine-grained analysis of clinical characteristics and their relation to deletion size is warranted.

### Limitations and future implications

First, our sample may encompass only an extreme subset of the PMS phenotype, which are the ones likeliest to have been diagnosed so far (younger and more affected individuals), and therefore may suffer from ascertainment bias. The profile may change in a couple of years when more systematic genetic testing for individuals with developmental delays exists. Future efforts should be put on harmonising measures across countries to build a common database for understanding this unique condition. Second, collecting all the questionnaires and other variables from the total sample proved to be difficult, due to time constraints and incomplete compliance with “off-site” measures. Further caveats are the lack of a standardised measure of cognitive ability (which also limits the interpretation of the results concerning verbal status), due to the difficulty of rigorously assessing a sample with a wide range of ages, severity of ID and limited language comprehension, and the lack of appropriate assessment tools, which hampers a more precise account of the syndrome herein discussed. To this effect, efforts are being made to review and discuss available instruments that may prove effective [45], since traditionally used methods are generally validated with normative samples. ADOS and ADI-R, for example, recognise as main limitations the assessment of autistic symptoms in ID or very young patients [23, 28, 46], and we acknowledge that the inclusion of clinical judgement regarding ASD symptoms would have provided a contextualized insight into the diagnostic prevalence of autism. However, the wide range of behavioural, clinical and genetic information we included depicts a clear picture of our sample and offers an unprecedented broad profile of a representative Spanish PMS cohort.

## Conclusions

This study adds to the available information on the clinical manifestation of PMS patients, who typically show impaired adaptive skills, speech acquisition delay or absence of language, and intellectual disability of variable degree, along with a number of medical and behavioural challenges, such as ASD, developmental regression and hyperactivity, some of which appear to correlate with deletion size. We emphasise the need for specific measurement tools that overcome current limitations and the importance of accurate psychiatric assessment to identify possible emotional dysregulations. Finding appropriate psychotherapeutic approaches and improving pharmacological treatments is crucial, as is understanding the response of this population to available medications and ascertaining whether they could benefit from specific and individualised treatments now under development. This will allow for personalised pharmacological and psychotherapeutic approaches. We highlight the need for longitudinal studies, to better describe PMS developmental trajectories and clinical courses, and better predict potential comorbidities of patients with Phelan-McDermid syndrome.

## Supplementary Information

**Additional file 1:.** Supplementary Figure 1. Hierarchical clustering dendrogram showing four clusters with the distribution of the included variables. Supplementary table 1. Sensory profile anomalies divided by language level. Supplementary table 2. Scores in behavioural questionnaires based on language level. Supplementary table 3 Medical complaints (parent interviews and medical records). Supplementary table 4. CIRS classification of reported events. Supplementary table 5. Differences in sample characteristics and 22q13 deletion size and trait correlation with deletion size. Supplementary table 6. Cluster number and clinical characteristics.

## Data Availability

The datasets used and/or analysed during the current study are available from the corresponding author on reasonable request.

## Abbreviations

PMS: Phelan-McDermid syndrome
ASD: Autism spectrum disorder
ID: Intellectual disability
M: Mean
SD: Standard deviation
ADOS-2: Autism Diagnostic Observation Schedule–2
ADI-R: Autism Diagnostic Interview–Revised
RBS-R: Repetitive Behaviours Scale–Revised
SRS: Social Responsiveness Scale
SP-2: Winnie Dunn Sensory Profile-2
CSS: Calibrated severity scores
VABS-3: Vineland Adaptive Behavior Scales-3
ABC-C: Aberrant Behavior Checklist–Community
CBCL: Child Behavior Checklist
SDQ: Strengths and Difficulties Questionnaire
CIRS: Cumulative Illness Rating Scale
FISH: Fluorescent in situ hybridization
MLPA: Multiplex ligation probe amplification
array-CGH/aCGH: Microarray-based comparative genome hybridization
