# Supplementary material for: Characterisation of the clinical phenotype in Phelan-McDermid syndrome
Source: J Neurodev Disord. 2021 Jul 10;13:26. doi: 10.1186/s11689-021-09370-5 (PMC8272382; doi:10.1186/s11689-021-09370-5)
Supplement: Supplementary file 1 — Additional file 1:. Supplementary Figure 1. Hierarchical clustering dendrogram showing four clusters with the distribution of the included variables. Supplementary table 1. Sensory profile anomalies divided by language level. Supplementary table 2. Scores in behavioural questionnaires based on language level. Supplementary table 3 Medical complaints (parent interviews and medical records). Supplementary table 4. CIRS classification of reported events. Supplementary table 5. Differences in sample characteristics and 22q13 deletion size and trait correlation with deletion size. Supplementary table 6. Cluster number and clinical characteristics. [file 11689_2021_9370_MOESM1_ESM.zip › Characterisation of clinical phenotype PMS_Suppmat_May2021.docx]

Characterisation of the clinical phenotype in Phelan-McDermid Syndrome

Mónica Burdeus-Olavarrieta*^1,2,3^, Antonia San José-Cáceres^1,2^, Alicia García-Alcón^1,2,4^, Javier González-Peñas^1,2,5^, Patricia Hernández-Jusdado^1^, Mara Parellada-Redondo^1,2,4^

^1^Department of Child and Adolescent Psychiatry, Institute of Psychiatry and Mental Health, Hospital General Universitario Gregorio Marañón, Madrid, Spain; ^2^IiSGM, Instituto de Investigación Sanitaria Gregorio Marañón, Madrid, Spain; ^3^School of Psychology, Universidad Autónoma, Madrid, Spain; ^4^School of Medicine, Universidad Complutense, Madrid, Spain; ^5^CIBERSAM, Centro de Investigación Biomédica en Red Salud Mental, Madrid, Spain.

*Corresponding author: Department of Child and Adolescent Psychiatry, Institute of Psychiatry and Mental Health, Hospital General Universitario Gregorio Marañón, Calle Ibiza 43, 28009, Madrid, Spain; Tel: (0034) 91 4265005; *E-mail address*: monica.burdeus@iisgm.com

# Supplementary materials

**Supplementary table 1**. Sensory profile anomalies divided by language level.

| Domain | Over percentile 80 | |
| --- | --- | --- |
|  | Verbal (*n*=10) | Minimally verbal (*n*=22) |
| Seeking | 50%  BCa 95% CI [30.0, 70.0] | 40.9%  BCa 95% CI [22.7, 59.1] |
| Avoiding | 70%  BCa 95% CI [50.0, 90.0] | 27.3%  BCa 95% CI [13.6, 41.5] |
| Sensitivity | 60.0%  BCa 95% CI [40.0, 80.0] | 50.0%  BCa 95% CI [36.4, 68.2] |
| Registration | 90.0%  BCa 95% CI [80.0, 100.0] | 77.3%  BCa 95% CI [59.1, 90.9] |
| Auditory | 20.0%  BCa 95% CI [0, 50.0] | 18.2%  BCa 95% CI [9.1, 31.8] |
| Visual | 0.0%  BCa 95% CI [-] | 18.2%  BCa 95% CI [4.5, 36.4] |
| Touch | 80.0%  BCa 95% CI [70.0, 90.0] | 68.2%  BCa 95% CI [50.0, 86.4] |
| Movement | 60.0%  BCa 95% CI [40.0, 80.0] | 68.2%  BCa 95% CI [50.0, 86.4] |
| Body position | 70.0%  BCa 95% CI [50.0, 90.0] | 72.7%  BCa 95% CI [54.5, 86.4] |
| Oral | 0.0%  BCa 95% CI [-] | 9.1%  BCa 95% CI [.0, 22.7] |
| Behavioural | 60.0%  BCa 95% CI [40.0, 80.0] | 45.5%  BCa 95% CI [27.3, 63.6] |
| Socio-Emotional | 70.0%  BCa 95% CI [50.0, 90.0] | 45.5%  BCa 95% CI [31.8, 63.6] |
| Attention | 70.0%  BCa 95% CI [50.0, 90.0] | 59.1%  BCa 95% CI [45.5, 77.3] |

Percentage of participants with scores classified as “more than others” and “much more than others”, corresponding to percentile at least above 80. BCa 95% confidence intervals computed via bootstrapping.

**Supplementary table 2.** Scores in behavioural questionnaires based on language level.

| Instrument | n | Minimally verbal | n | Verbal | Comparison |
| --- | --- | --- | --- | --- | --- |
| SDQ Total | 23 | 14.96(*5.02*)[6-25]  BCa 95% CI [12.74, 17.04] | 10 | 15.80(*6.68*)[2-23]  BCa 95% CI [11.27, 19.50] | *t*(31)=-.401, 95% CI [-5.13, 3.44], *p=*.691 |
| SDQ Emotional |  | 1.48(*1.20*)[0-4]  BCa 95% CI [1.04, 1.91] |  | 2.40(*1.65*)[0-5]  BCa 95% CI [1.50, 3.34] | U=74.500, Z=-1.654, *p=*.098 |
| SDQ Conduct |  | 1.43(*1.20*)[0-4]  BCa 95% CI [1.00, 1.87] |  | 2.20(*1.40*)[0-4]  BCa 95% CI [1.50, 3.00] | U=80.00, Z=-1.422, *p*=*.155* |
| SDQ Hyperactivity |  | 7.00(*2.43*)[3-10]  BCa 95% CI [6.08, 7.86] |  | 7.00(*2.87*)[2-10]  BCa 95% CI [5.50, 8.44] | *t*(31)=.000, 95% CI [-1.98,1.98], *p=*1.000 |
| SDQ Peer relations |  | 5.04(*2.10*)[2-9]  BCa 95% CI [4.22, 5.83] |  | 4.20(*2.49*)[0-8]  BCa 95% CI [2.80, 5.70] | U=95.500, Z=-.778, *p=*.436 |
| SDQ Prosocial |  | 2.87(*2.51*)[0-9]  BCa 95% CI [2.13, 3.70] |  | 5.40(*2.72*)[1-8]  BCa 95% CI [4.00, 6.80] | U=53.500, Z=-2.443, *p=*.015 |
| CBCL 1.5-5 Emot. reactive | 10 | 5.50(*3.63*)[0-11]  BCa 95% CI [3.40, 7.80] |  | n/a | n/a |
| CBCL 1.5-5 Anxious/Dep |  | 2.80(*2.10*)[0-6]  BCa 95% CI [1.50, 4.00] |  |  |  |
| CBCL 1.5-5 Somatic |  | 1.70(*1.49*)[0-4] BCa 95% CI [.80, 2.60] |  |  |  |
| CBCL 1.5-5 Withdrawn |  | 7.40(*3.92*)[2-12] BCa 95% CI [5.10, 9.90] |  |  |  |
| CBCL 1.5-5 Sleep |  | 4.10(*2.96*)[0-10] BCa 95% CI [2.30, 5.90] |  |  |  |
| CBCL 1.5-5 Attention |  | 6.10(*1.79*)[4-9]  BCa 95% CI [5.00, 7.20] |  |  |  |
| CBCL 1.5-5 Aggresive |  | 15.20(*7.41*)[4-26]  BCa 95% CI [10.60, 19.80] |  |  |  |
| CBCL 6-18 Anxious/Dep | 17 | 2.64(*1.97*)[0-6]  BCa 95% CI [1.82, 3.54] | 12 | 3.67(*1.83*)[1-7]  BCa 95% CI [2.67, 4.75] | U=75.000, Z=-1.218, *p=*.223  η^2^*=*0.053 |
| CBCL 6-18 Withdrawn/Dep |  | 3.24(*2.33*)[0-8]  BCa 95% CI [2.24, 4.18] |  | 3.00(*2.09*)[0-7]  BCa 95% CI [1.92, 4.08] | *t*(27)=.279, 95% CI [-1.49, 1.97], *p=*.782 |
| CBCL 6-18 Somatic |  | 2.88(*2.40*)[0-9]  BCa 95% CI [1.82, 4.06] |  | 3.50(*2.84*)[0-9]  BCa 95% CI [2.08, 5.08] | *t*(27)=-.633, 95% CI [-2.62, 1.38], *p=*.335 |
| CBCL 6-18 Social |  | 6.94(*2.63*)[0-11]  BCa 95% CI [5.76, 8.06] |  | 8.08(*4.01*)[2-15]  BCa 95% CI [6.08, 10.15] | U=84.000, Z=-.801, *p=*.423  η^2^*=*0.023 |
| CBCL 6-18 Thought |  | 4.65(*3.06*)[0-11]  BCa 95% CI [3.08, 6.12] |  | 5.92(*3.96*)[0-13]  BCa 95% CI [3.75, 8.19] | *t*(27)=-.974, 95% CI [-3.94, 1.41], *p=*.339 |
| CBCL 6-18 Attention |  | 10.77(*4.10*)[3-17]  BCa 95% CI [8.60, 12.65] |  | 10.58(*3.85*)[1-16]  BCa 95% CI [8.33, 12.58] | *t*(27)=.120, 95% CI [-2.91, 3.28], *p=*.905 |
| CBCL 6-18 Rule breaking |  | 3.12(*2.71*)[0-10]  BCa 95% CI [1.94, 4.29] |  | 2.17(*1.90*)[0-6]  BCa 95% CI [1.17, 3.25] | *t*(27)=1.045, 95% CI [-.92, 2.82], *p=*.305 |
| CBCL 6-18 Aggresive |  | 7.18(*4.73*)[0-20]  BCa 95% CI [5.06, 9.47] |  | 9.42(*5.16*)[0-15]  BCa 95% CI [6.00, 12.42] | *t*(27)=-1.210, 95% CI [-6.04, 1.56], *p=*.237 |
| ABC Agitation/ Irritability | 28 | 7.46(*6.61)*[0-22]  BCa 95% CI [5.07, 10.18] | 16 | 8.50(*7.40*)[0-22]  BCa 95% CI [5.00, 11.75] | U=218.000, Z=-.147, *p=*.883 |
| ABC Social Withdrawal |  | 8.46(*7.64*)[0-27]  BCa 95% CI [5.52, 11.86] |  | 7.44(*6.71*)[0-23]  BCa 95% CI [4.56, 10.81] | U=213.500, Z=-.257, *p=*.797 |
| ABC Stereotyped Behavior |  | 5.79(*5.83*)[0-18]  BCa 95% CI [3.46, 8.30] |  | 2.25 (*2.96*)[0-10]  BCa 95% CI [1.06, 3.63] | U=145.000, Z=-1.962, *p=*.050 |
| ABC Hyperactivity |  | 14.29(*10.1*)[0-39]  BCa 95% CI [10.75, 18.41] |  | 10.56(*10.17*)[0-25]  BCa 95% CI [5.80, 15.85] | *t*(42)=1.173, 95% CI [-2.68, 10.13], *p=*.247 |
| ABC Repetitive Speech |  | .61(*.95*)[0-3]  BCa 95% CI [.28, 1.00] |  | 3.06(*3.11*)[0-10]  BCa 95% CI [1.75, 4.51] | U=93.500, Z=-3.393, *p=*.001 |

**SDQ:** Strengths and difficulties questionnaire. **CBCL:** Child Behavior Checklist. Version for children aged 1.5-5 years only included non-verbal participants in our sample. **ABC:** Aberrant Behavior Checklist. | BCa 95% confidence intervals of the means computed via bootstrapping. Mann-Whitney-U tests performed for non-normally distributed variables.

**Supplementary table 3.** Medical complaints (parent interviews and medical records).

| Medical comorbidities | N | Percentage |
| --- | --- | --- |
| Musculoskeletal/Hypotonia | 53 | 88.3% |
| Recurrent infections (childhood) | 45 | 75.0% |
| Lower gastrointestinal complaints | 35 | 58.3% |
| Neurologic (includes epilepsy/seizures) | 33 | 55.0% |
| Ophthalmic/Otorhinolaryngology (ETN) | 32 | 53.3% |
| Upper gastrointestinal complaints | 30 | 50.0% |
| Respiratory | 30 | 50.0% |
| Food intolerances | 24 | 40.0% |
| Sleep difficulties | 19 | 31.7% |
| Renal | 18 | 30.0% |
| Vascular | 18 | 30.0% |
| Skin complaints | 16 | 26.7% |
| Hematologic | 15 | 25.0% |
| Cardiac | 14 | 23.3% |
| Epilepsy/Febrile seizures | 13 | 21.7% |
| Genitourinary | 11 | 18.3% |
| Endocrine-metabolic | 10 | 16.7% |

**Supplementary table 4**. CIRS classification of reported events.

| System | 1 Mild problem | 2 Moderate problem | 3 Severe problem |
| --- | --- | --- | --- |
| 1-Cardiac | Neonatal heart murmur; occasional tachycardia | Structural cardiac anomalies like bifurcation of vena cava, neonatal ductus, aortic dilation and anomalous pulmonary vein drainage | - |
| 2-Vascular | Occasional swelling of ankles; hyper- and hypohidrosis; altered temperature regulation; tendency to cold limbs | Lymphedema; very frequent swelling of limbs and/or very frequent cold limbs | - |
| 3-Hematological | Low iron levels | - | - |
| 4-Respiratory | Frequent bronchiolitis and/or other minor upper or lower respiratory tract infections | Congenital tracheomalacia; frequent apnea; frequent pneumonias; only one working lung | - |
| 5-Ophthalmic and otorhino-  laryngology | Recurrent otitis; tympanic drainages; mucus excess in inner ears; adenoidectomy; recurrent laryngitis; hypermetropia; strabismus; astigmatism | Bilateral hearing loss | - |
| 6-Upper gastrointestinal | Gastroesophageal reflux | Reflux operation; ileitis with recurrent vomits | Esophageal atresia |
| 7-Lower gastrointestinal | Constipation; Diarrhoea | Severe/chronic constipation; severe/chronic diarrhoea; gastric/bowel distension | Anal atresia |
| 8-Hepatic and pancreatic | Elevations of transaminases in remission | - | - |
| 9-Renal | Mild asymmetry; horseshoe kidney; vesicoureteral reflux; microcysts; scarred kidneys | Ectopic kidney; duplex kidney; pyeloureteral stenosis; renal tubular acidosis; hydronephrosis with vesicoureteral reflux; reflux operation | Kidney dysplasia and metabolic acidosis; acute kidney failure |
| 10-Genitourinary | Urinary tract infections; overactive bladder; cryptorchidism; balanitis; hypospadias; megacystis | Frequent urinary tract infections and double vagina; adhered ovaries operation; recurrent urinary tract infections | - |
| 11-Musculoskeletal | Hypotonia; hypertonia; scoliosis; joint hyperlaxity | Severe hypotonia; hypotonia with spasticity or joint hyperlaxity; hypotonia with bone anomalies that affect walking and may require splints or operations, like kyphosis, scoliosis, internal hip rotation with intoeing, genu varum, ankle valgus, claw feet, flat feet, equinovarus feet; pectus excavatum | Unable to walk (by age 4) |
| 12-Neurological | Febrile seizures; paroxysmal or subclinical EEG anomalies; asymptomatic structural anomalies like arachnoid cysts or mild white matter atrophy or gliosis; benign hydrocephalus | Epilepsy and recurrent febrile seizures; cerebellar vermis hypoplasia; corpus callosum hypoplasia or atrophy and ventriculomegaly; hydrocephalus operation; megacisterna magna with ventriculomegaly and ischemic injuries; arachnoid cysts with further anomalies like Rathke's cleft cyst and empty sella syndrome; periventricular atrophy; cerebral atrophy with lumps | Ischemic stroke with hemiparesis; periventricular leukomalacia |
| 13-Endocrine, metabolic, breast | Neonatal thelarche; breast asymmetry and low weight; short stature; low prolactin with subclinical hypothyroidism; tendency to hyperthyroidism; accelerated growth; low D vitamin | Precocious puberty with regular hormone treatment; growth hormone treatment; treated hypothyroidism | - |
| 14-Psychiatric | Motor restlessness | Hyperactivity or severe motor restlessness and attention deficit; ADHD diagnosis; frequent sleep disturbances; disruptive or aggressive behaviour; alternation of irritability and apathy | Clinical ASD diagnosis; mood oscillations with hypomania and sleep disturbances; bipolar disorder |

Reported medical comorbidities categorised within CIRS severity levels. Medical history was explored, results are retrospective and encompass all lifetime. Although some findings were severe and/or chronic, none was categorised as “4-Extremely severe problem” because treatment options were available and no prognosis was grave at the time of the assessment.

**Supplementary table 5.** Differences in sample characteristics and 22q13 deletion size and trait correlation with deletion size.

| Variable | N | 22q13 deletion size (Mb) | Comparison |
| --- | --- | --- | --- |
| Gender  Male  Female | 27  26 | 3.20 (*2.66*) [0.01-8.53]  3.05 (*2.58*) [0.04-7.76] | *t*(51)=.205, 95% CI [-1.30, 1.59], *p=*.838 |
| Language(≥3yo)*  No language  Words  Sentences  Fluid | 28  5  8  6 | 3.43 (*2.65*) [0.01-8.24]  2.95 (*1.78*) [0.02-4.55]  2.47 (*1.44*) [0.28-4.96]  0.62 (*1.29*) [0.02-3.25] | *F*(3,49)=3.012, *p=*.039 |
| Regression  Yes  No | 22  31 | 2.57 (*2.27*) [0.01-7.42]  3.52 (*2.77*) [0.02-8.53] | *t*(51)=1.324, 95% CI [-.49, 2.39], *p=*.191 |
| Motor development  Delayed walking: Yes  Delayed walking: No | 28  23 | 4.38 (*2.14*) [0.28-8.24]  1.17 (*1.42*) [0.01-4.20] | *t*(49)=-6.147, 95% CI [-4.25, -2.16], *p*<.001 |
| Trait correlation  ADOS-SA-CSS  ADOS-RRB-CSS  ADOS-Total-CSS  ADI-R A  ADI-R B  ADI-R C  VABS Communication  VABS Daily Living  VABS Socialization  VABS Total Adaptive | 42  43  41 | *r_s_*(40)=-.126  *r_s_*(40)=.119  *r_s_*(40)=-.109  *r_s_*(41)=.306  *r_s_*(41)=.236  *r*(41)=-.089  *r_s_*(39)=-.144  *r*(39)=-.147  *r*(39)=-.196  *r*(39)=-.196 | BCa 95% CI [-.460, .200] *p=*.428  BCa 95% CI [-.460, .200] *p=*.428  BCa 95% CI [-.444, .228] *p=*.493  BCa 95% CI [-.011, .579] *p=*.046  BCa 95% CI [-.044, .479] *p=*.128  BCa 95% CI [-.375, .197] *p=*.569  BCa 95% CI [-.479, .191] *p=*.561  BCa 95% CI [-.503, .243] *p=*.358  BCa 95% CI [-.539, .186] *p=*.219  BCa 95% CI [-.546, .194] *p=*.220 |

Mb=megabases. Genetic information available for 53 subjects. ***Language:** One-way ANOVA used *(F*=3.012, *p=*.039; Post-hoc Tukey HSD: *“*No language” > “Fluid” 95% CI [.24, 6.02], *p=*.029*).* **Trait correlation:** Spearman’s rho reported for non-normally distributed variables. BCa 95% CI computed via bootstrapping for correlation coefficients.

**Supplementary table 6.** Cluster number and clinical characteristics.

| Variable | Cluster | | | |
| --- | --- | --- | --- | --- |
|  | C1 (n=11) | C2 (n=6) | C3 (n=10) | C4 (n=15) |
| Deletion size (Mb) | M=4.16*(1.70)*  [2.18-8.24] | M=5.51*(1.47)*  [4.10-7.42] | M=2.60*(.96)*  [1.20-4.27] | M=.35*(.46)*  [.01-1.40] |
| ADOS-2 total CSS score | Median=3  [2-4] | Median=8.5  [6-10] | Median=6  [6-7] | Median=6  [3-9] |
| Delayed walking: Yes | 9 (81.8%) | 5 (83.3%) | 8 (80%) | 1 (6.7%) |
| Regression: Yes | 2 (18.2%) | 5 (83.3%) | 4 (40%) | 9 (60%) |
| Language: Yes | 3 (27.3%) | 1 (16.7%) | 3 (30%) | 6 (40%) |
| Sex | 3M (27.3%) | 3M (50%) | 9M (90%) | 6M (40%) |

Cluster number and main clinical characteristics of each cluster.

**Supplementary figure 1.**  Hierarchical clustering dendrogram.

[ Please insert Supplementary Figure 1 here ]

**Supp Fig 1.** Hierarchical clustering dendrogram showing four clusters with the distribution of the included variables.
