# Supplementary material for: Characterisation of the clinical phenotype in Phelan-McDermid syndrome
Source: J Neurodev Disord. 2021 Jul 10;13:26. doi: 10.1186/s11689-021-09370-5 (PMC8272382; doi:10.1186/s11689-021-09370-5)
Supplement: Supplementary file 1 — Additional file 1:. Supplementary Figure 1. Hierarchical clustering dendrogram showing four clusters with the distribution of the included variables. Supplementary table 1. Sensory profile anomalies divided by language level. Supplementary table 2. Scores in behavioural questionnaires based on language level. Supplementary table 3 Medical complaints (parent interviews and medical records). Supplementary table 4. CIRS classification of reported events. Supplementary table 5. Differences in sample characteristics and 22q13 deletion size and trait correlation with deletion size. Supplementary table 6. Cluster number and clinical characteristics. [file Characterisation PMS_ Supp fig1 Cluster_mar21.pdf › Characterisation PMS_ Supp fig1 Cluster_mar21.pdf]

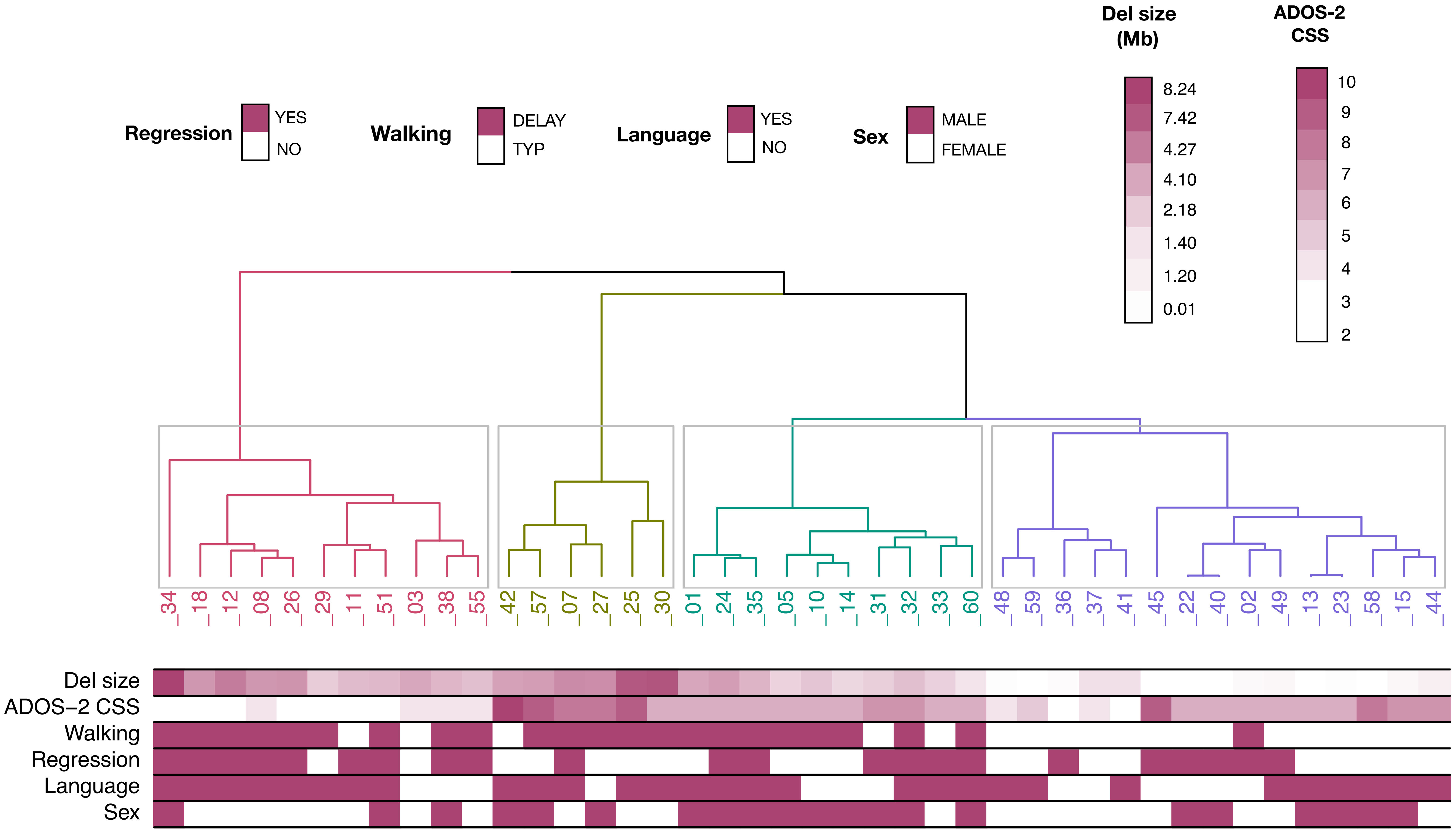

Regression
YES
NO
Walking
DELAY
TYP
Language
YES
NO
Sex
MALE
FEMALE
Del size (Mb)
8.24
7.42
4.27
4.10
2.18
1.40
1.20
0.01
ADOS-2 CSS
10
9
8
7
6
5
4
3
2
_34 _18 _12 _08 _26 _29 _11 _51 _03 _38 _55
_42 _57 _07 _27 _25 _30
_01 _24 _35 _05 _10 _14 _31 _32 _33 _60
_48 _59 _36 _37 _41 _45 _22 _40 _02 _49 _13 _23 _58 _15 _44
Del size
ADOS–2 CSS
Walking
Regression
Language
Sex
